# To what extent is research on infrahumanization confounded by intergroup preference?

**DOI:** 10.1098/rsos.241348

**Published:** 2025-04-16

**Authors:** Carl Bunce, Adam Eggleston, Robert Brennan, Harriet Over

**Affiliations:** ^1^ Department of Psychology, University of York, York, Yorkshire, UK; ^2^ School of Psychology and Clinical Language Sciences, University of Reading, Reading, UK; ^3^ York St John University, York, UK; ^4^ The Institute of Future, Media, Democracy and Society, Dublin City University, Dublin, Ireland

**Keywords:** dehumanization, infrahumanization, intergroup bias, scientific methods, open science, emotions

## Abstract

The most prominent social psychological account of dehumanization, infrahumanization theory, argues outgroups are dehumanized to the extent they are denied uniquely human emotions. Recent critiques have identified a confound in previous research whereby uniquely human emotions used as stimuli tend to be more prosocial than the emotions shared with other species. Consequently, apparent evidence for subtle dehumanization may be better explained by intergroup preference. While there is growing appreciation that some studies are confounded this way, the extent of this problem has proved controversial. To gauge prevalence of the confound, we systematically reviewed the infrahumanization literature and extracted all emotion terms used. Participants rated the extent to which these emotions appeared unique to humans and prosocial. From these data, we calculated the percentage of studies that confound humanness with prosociality. In the 10 most cited papers, 95.5% of reported studies were confounded in the predicted direction. Across all 152 studies, 79.6% showed the same issue. These findings point to a pervasive methodological problem, impacting our understanding of discrimination and the reliability of social psychological data. To facilitate progress moving forward, we introduce a freely accessible tool, powered by our emotion rating database, to help researchers generate rigorously controlled stimulus sets.

## 1. Introduction

The dehumanization hypothesis holds that members of disadvantaged social groups are often vulnerable to harm because they are perceived to be less human than the ingroup [1,2]. When groups are blatantly dehumanized, they are thought to be excluded from the human category and, as a result, placed at risk of extreme harm such as genocide, rape and torture [1–4]. Support for this claim comes primarily from the historical record, particularly from analyses of propaganda. During the Holocaust, Nazi propaganda regularly compared Jewish people with cockroaches and rats [2,5]. During the 1994 genocide in Rwanda, Hutu propaganda often compared Tutsis with snakes [6].

Social psychological theories of dehumanization maintain that dehumanization exists along a continuum from blatant to subtle forms [7]. In subtle forms of dehumanization, outgroup members are thought to be considered somewhat ‘less human’ than ingroup members and to possess uniquely human emotions, traits and other mental states to a lesser extent than the ingroup [8–11]. Empirical support for subtle forms of dehumanization comes primarily from laboratory-based research using experimental and correlational designs. Multiple empirical studies have reported that when groups are subtly dehumanized, they are less likely to be the recipients of prosocial behaviour [12–14].

Among the most prominent social psychological theories of dehumanization is infrahumanization theory [11]. According to infrahumanization theory, outgroup members are often subtly dehumanized by being denied uniquely human, or secondary, emotions. In typical studies testing this theory, participants are presented with secondary emotions that are perceived to be uniquely human (e.g. remorse, guilt) and primary emotions that are perceived to be shared with other species (e.g. fear, anger). They are then asked how characteristic these different emotional experiences are of the ingroup and the outgroup. To the extent that outgroups are denied secondary emotions, they are thought to be dehumanized [11,15–18]. Infrahumanization has been reported across a range of intergroup contexts [15,19] and has been measured with implicit as well as explicit tasks [17,20].

Of key importance to this theory is the claim that infrahumanization is distinct from intergroup preference, a general bias whereby participants may assign more favourable response options to their own group and/or more negative response options to the outgroup. Infrahumanization is assumed to be distinguishable from intergroup preference because participants ascribe secondary emotions that are both positive to experience (e.g. hope, compassion) and negative to experience (e.g. guilt, remorse) more strongly to the ingroup. Haslam & Stratemeyer [21, p. 25] emphasize that, ‘*Seeing someone as lacking human qualities is not the same as derogating them because ‘human’ is not synonymous with ‘good*’’. Thus, as articulated by Leyens *et al*. [11, p. 401], ‘*If the attribution of secondary emotions to the ingroup reflected a mere positivity effect, it would lose its interest and originality…*’.

Recently, Enock and colleagues have argued that evidence claiming to distinguish infrahumanization from intergroup preference may be less convincing than it first appears [22,23]. The authors highlight that an aspect of emotions largely overlooked in the literature is their inherent social nature. Emotions vary not only in the extent to which they are judged to be positive or negative to personally experience (valence), but also in the extent to which they are judged to be prosocial and antisocial when expressed by others. Prosocial emotions can be defined as those that reflect a tendency to act in ways that benefit or show consideration for others, typically expressed by individuals we consider kind. Antisocial emotions, on the other hand, reflect a tendency to act in ways that promote harm or demonstrate a lack of concern for others, typically expressed by individuals we consider unkind.

Crucially, this prosociality dimension of emotions does not always align with—and in many cases, conflicts with—the valence dimension. For instance, Enock *et al*. [22] point out that although emotions such as guilt and remorse are negative to experience, they are not antisocial in character. Rather, they are thought to foster prosocial, reparative responses, and individuals who display them following wrongdoing tend to be viewed more positively than those who do not [22,23]. There is thus a potentially critical confound in previous studies of infrahumanization whereby the secondary emotions included in stimulus sets are more prosocial than the primary emotions. As a result, what appears to be a subtle process of dehumanization may, in fact, be better explained in terms of intergroup preference: that is, a bias to attribute more kind or unkind emotions to ingroups and outgroups, respectively.

Empirically addressing this critique, Enock and colleagues [22] recently re-examined the central predictions of infrahumanization theory while controlling for emotion prosociality by comparing attribution of secondary emotions that are perceived as socially desirable, such as optimism and humility, with attribution of secondary emotions that are perceived as socially undesirable, such as arrogance and contempt. Contrary to the predictions of infrahumanization theory, when emotion prosociality was controlled for, outgroup members were not denied secondary emotions. Rather, outgroup members were attributed prosocial emotions to a lesser extent than ingroup members, but attributed antisocial emotions to a greater extent than ingroup members regardless of whether or not these emotions were unique to humans. In further work, Enock & Over [23] re-examined the behavioural consequences of infrahumanization and found that there was no relationship between secondary emotion attribution and prosocial behaviour when the prosociality of the emotions was controlled for.

At present, the number of studies affected by the prosociality confound is unclear. Enock and colleagues tested seven intergroup contexts including political groups, religious groups and minimal groups created in the laboratory [22]. They suggest that this may be a far-ranging problem that affects much of the literature in this area (see also [23,24]). Vaes [25, p. 2], on the other hand, has argued that ‘*the number of studies that has demonstrated outgroup dehumanization over and above intergroup prejudice and outgroup derogation are much higher than Enock et al. [22] would like to admit*’. Understanding which studies are affected by a confound with prosociality and which are not is a crucial priority for the field moving forward. Without this understanding, it is impossible to determine the extent to which subtle dehumanization of this form contributes to discrimination.

Here, we seek to understand the scale of the confound in previous research and propose constructive developments for the field moving forward. In order to do this, we systematically search the literature for as many studies as possible that have experimentally manipulated the attribution of primary and secondary emotions in order to measure infrahumanization and its behavioural consequences. From these studies, we extract all of the emotion terms used as stimuli (stage 1). We then ask a large sample of participants (total *n* = 600) to rate these emotion terms on three variables crucial to understanding infrahumanization theory: (i) the extent to which they are uniquely human versus shared with other species; (ii) the extent to which they are generally positive to experience versus negative to experience; and (iii) the extent to which they are generally prosocial versus antisocial (stage 2). Next, we use these ratings to understand which previous manipulations of perceived humanness are affected by a confound with prosociality and which are not (stage 3). In order to do this, we extract the stimuli used for all eligible studies and compare whether the secondary emotions were rated as significantly more prosocial than were the primary emotions. Following this, as a way of demonstrating the impact of improperly controlled stimulus sets in driving results resembling infrahumanization, we conduct a typical infrahumanization study using the stimulus sets we previously identified as being most and least confounded on the dimension of prosociality. We then assess whether the presence of the confound significantly influences the extent to which there appears to be evidence of outgroup infrahumanization (stage 4).

In conducting this research, we are not trying to determine whether infrahumanization theory is correct or incorrect, but rather to identify which studies may need to be reassessed in light of a confound between humanness and prosociality that has been demonstrated in previous work [22,23]. We close by introducing an open-source tool we have developed with the intention of assisting researchers in generating controlled stimulus sets for their future investigations.

## 2. Transparency and openness

We pre-registered each stage of our project: stage 1—our systematic search of the literature (https://osf.io/6z5sp/?view_only = 733852998a734680b44500c41d7f6ec3); stage 2—data collection (https://osf.io/g6vsh/?view_only = aa460a21119b4ceeace05ba5e95b27d4); stage 3—analysis (https://osf.io/byszq/?view_only = 81a4ee381c684dcc8923872ddf4f8087); and stage 4—infrahumanization experiment (https://aspredicted.org/TTP_BYC). All data supporting each stage, including exclusions, participant ratings and the Emotion Stimuli Selector tool, can be found on the Open Science Framework (OSF; https://osf.io/zs2a6/; [26]) and Dryad [27].

## 3. Stage 1: collating the emotion terms used in previous research

### 3.1. Method

#### 3.1.1. Study selection

In stage 1, we searched the literature for as many studies as possible that have experimentally manipulated the attribution of primary and secondary emotions in order to measure infrahumanization and its behavioural consequences. We took a systematic approach to this search. A full outline of our protocol for each stage can be found using the links above. Stage 1 outlines our eligibility criteria and search strategies. In summary, we included all study designs where the perceived humanness of the emotions presented to participants was manipulated in order to create categorical variables of secondary emotions (those perceived to be unique to humans) and primary emotions (those perceived to be shared with other species [10,11]). While not all studies on infrahumanization use this design [25,28], we chose to focus on it because it is by far the most common design within the literature and because it has been most directly critiqued [22]. Of these studies, only those studies investigating and citing infrahumanization as it was originally conceived as differences in emotion attribution [10,11] were selected. Following these guidelines, we did not include studies that used other terms related to animal and humans such as ‘mongrel’ and ‘citizen’ (e.g. [29]) or studies that related to other theories of dehumanization such as the Dual Model which focuses on the attribution of character traits to different groups rather than emotions [9]. For practical reasons, only studies reported in English were included. Editorials, notes and news briefings were excluded. Included studies were limited to those published between 2000 and the day we began the literature search (29 November 2022). We incorporated this limit because the concept of infrahumanization was first introduced into the psychological literature in 2000 [10].

**Figure 1.**
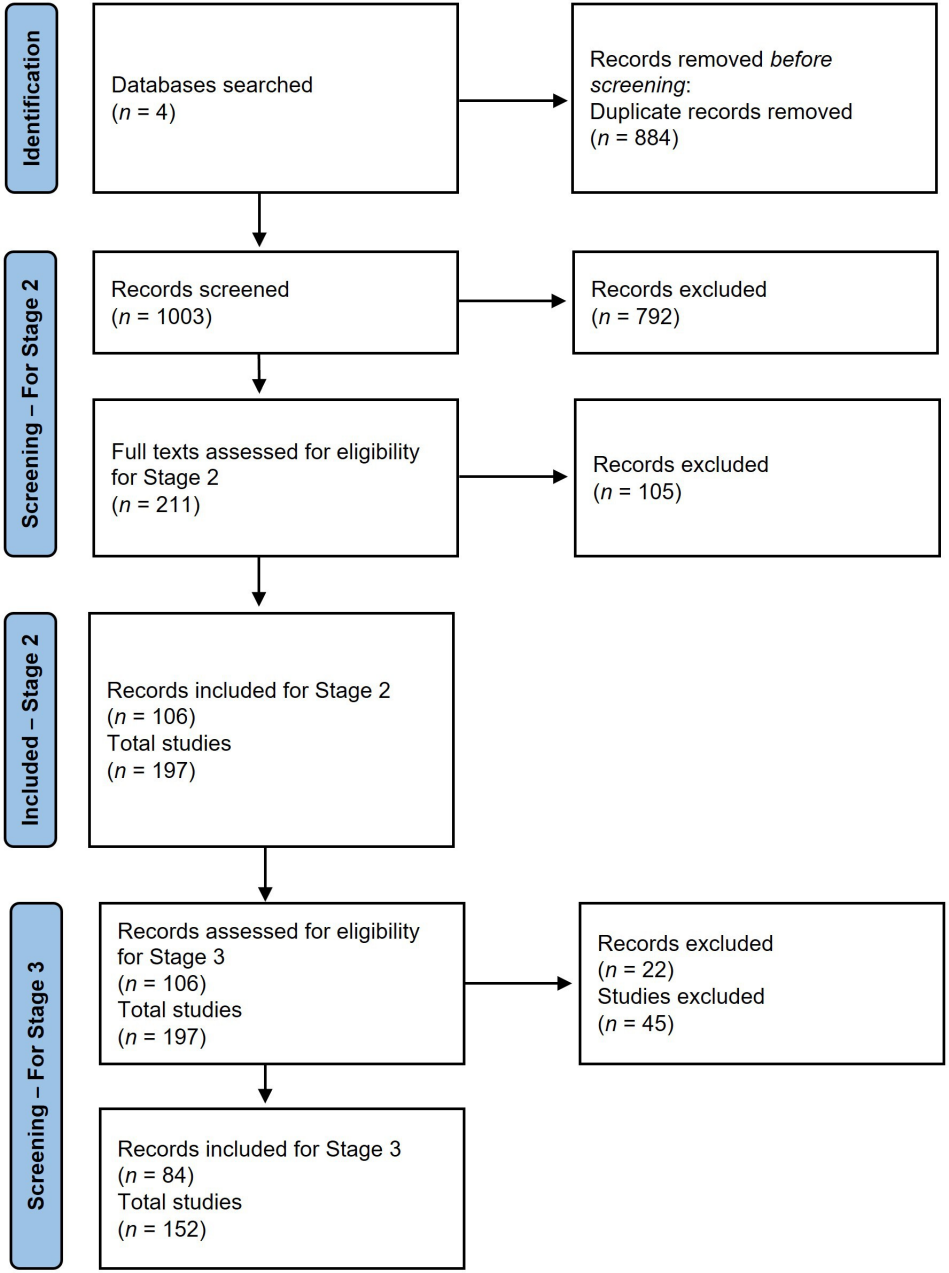
PRISMA flowchart of study inclusion and exclusion at each stage of the experimental procedure: 84 papers were included in the final selection which corresponds to 152 individual studies.

Searches were undertaken in a collection of online databases to identify eligible published studies. Four key databases were searched: PsycINFO, Science Citation Index, Social Science Citation Index and Scopus. Alongside database searches, we searched for published studies by key researchers in the field (e.g. Leyens, Vaes, Paladino, Viki, Demoulin) and the reference list of key review articles [9,30,31]. The results of each search were downloaded in a tagged format to simplify the uploading of all references into bibliographical software (EndNote). In cases where that was not possible, relevant documents were uploaded manually. Results were deduplicated using EndNote v.20 and then again in Covidence. The deduplication process identified overlapping references across the databases searched to allow for only unique entries to be assessed for eligibility. The number of duplicates removed and exclusions at each stage can be seen in figure 1.

Initial record selection, based on title and abstract, was undertaken by two reviewers in Covidence. Obvious false positives (such as medical studies, non-empirical work and work covering topics clearly unconnected to infrahumanization) were excluded in this initial stage. If there was ambiguity in a record’s eligibility, then the full text was assessed. A third reviewer was used to resolve any disagreements. Following this stage, the full text of all potentially relevant studies was obtained and reviewed.

In the second stage of assessment, each full text was reviewed by two reviewers based on pre-determined eligibility criteria. All decisions at this stage were saved in Covidence and a full list of excluded studies and the reason for exclusion can be found on OSF/Dryad [26,27]. Again, any disagreements were resolved by a third reviewer.

#### 3.1.2. Data extraction

We were interested in extracting secondary emotions and primary emotions used in all studies included in our final sample. For studies where emotion valence (that is, what the emotion feels like to experience) was also reported, that information was extracted as well. For all eligible studies, data were deposited in a spreadsheet and a second reviewer checked that all emotion terms extracted from eligible studies were accurate. The few disagreements were discussed and resolved with the help of a third reviewer. In cases where only a subset of the emotion terms presented to participants were reported or where terms were reported but not their perceived humanness (primary or secondary), the corresponding author was contacted for the full list of emotion terms used. We allowed three weeks from first contact to receive a response. If authors did not or could not respond, that study was excluded from stage 3 but the available emotion terms were still rated by participants during the emotion rating task (stage 2) to allow our emotion database to be as inclusive and varied as possible.

### 3.2. Results

A total of 1003 records were screened. Of these, 792 records were excluded for obviously not meeting the inclusion criteria based on title and abstract screening. The full texts of the remaining 211 papers were assessed leading to the exclusion of a further 105 papers for not meeting our pre-registered eligibility criteria. From this list of studies, we created a list of all unique emotion terms used across the 197 studies found eligible in the 106 included records. A final 22 papers were excluded from analysis in stage 3. Reasons for this include not reporting all emotion terms or not providing them on request, only using secondary emotions, and not investigating infrahumanization in the context of human groups. This final round of exclusions included six studies from five papers that were identified as not meeting our eligibility criteria following the pre-registration of our stage 3 analysis plan.^1^ This resulted in a total of 84 eligible papers for our final analysis. Many of these 84 papers incorporated multiple experiments, leading to a total of 152 studies to be assessed for evidence of a confound. A breakdown of the process at each stage can be found in figure 1.

## 4. Stage 2: creating an emotion database

In stage 2, our goal was to create a database of emotions rated on the three dimensions that are crucial for understanding infrahumanization theory: (i) the extent to which they are perceived as uniquely human versus shared with other species; (ii) the extent to which they are generally perceived as positive to experience versus negative to experience; and (iii) the extent to which they are generally perceived as prosocial versus antisocial.

We had two goals in mind when creating this database. First, we sought to understand how the stimuli used in previous research vary in terms of perceived humanness and prosociality. In doing so, we hoped to understand the extent to which researchers have inadvertently confounded humanness with prosociality in previous research. Second, we sought to create a valuable resource for the field. A large database of emotions rated on these dimensions will allow experimenters to choose appropriately controlled stimuli in the future.

In order to make our database of emotion terms as large, and thus as useful to the field, as possible we supplemented the list of terms we extracted during stage 1 with emotions found in other relevant work (e.g. [36]). This also allowed us to incorporate more antisocial emotions into the database, which we hypothesized were lacking in much previous research on infrahumanization.

We asked 600 participants to rate 250 emotions along one of the three dimensions outlined above. In order to protect against carryover effects, three unique sets of participants were asked to rate the 250 emotions, one for each of the three dimensions outlined above.

### 4.1. Method

#### 4.1.1. Participants

Six hundred adult participants who speak English as their first language rated the entire set of emotions on one of three questions (200 per question set). The procedure was approved by the University of York Department of Psychology’s Ethics Committee and all methods were performed in accordance with the committee’s guidelines and performed in accordance with the Declaration of Helsinki. All participants were recruited via the online platform Prolific (https://www.prolific.com), tested via Qualtrics (https://www.qualtrics.com) and reimbursed at an hourly rate of £9.

*Humanness ratings*: two hundred participants rated the stimuli for perceived humanness. Seven participants were excluded and replaced—five owing to failing 50% or more of the attention checks and two for reporting that English was not their first language. Of the 200 participants (*M*_age_ = 38.79, s.d._age_ = 12.87), 83 used female pronouns, 110 male pronouns, four non-binary pronouns and three preferred not to say.

*Prosociality ratings*: two hundred participants rated the stimuli for perceived prosociality. One participant was excluded and replaced owing to failing 50% or more of the attention checks and two were excluded for reporting that English was not their first language. Of the 200 participants (*M*_age_ = 38.95, s.d._age_ = 14.56), 116 used female pronouns, 73 male pronouns, eight non-binary pronouns and three preferred not to say.

*Valence ratings*: two hundred participants rated the stimuli for how positive they were to experience. One participant was excluded and replaced owing to failing 50% or more of the attention checks and one was excluded for reporting that English was not their first language. Of the 200 included (*M*_age_ = 37.42, s.d._age_ = 11.34), 73 used female pronouns, 122 male pronouns, three non-binary pronouns and two preferred not to say.

#### 4.1.2. Design and materials

The total database consisted of 250 emotion terms, a full list of which can be found in the electronic supplementary material. Participants were asked to rate all of the emotion terms on one of three dimensions following procedures similar to those used in other research (e.g. [22]). In some cases, different researchers used variants of the same emotion term in their papers. For example, whereas some researchers used the term ‘nostalgic’, others used the term ‘nostalgia’. For completeness, we included all variants of each emotion term in the database. This creates some repetitions in the emotion terms. However, it meant that, when seeking to understand whether or not stimuli were confounded in stage 3, we could use exactly the same terms as used in previous research and so fairly assess the extent of the confound.

The humanness scale asked, ‘*Using the slider, please indicate how much the emotion in each of the following questions is experienced by humans compared to other species (i.e. is this emotion unique to humans?)*’. The bottom end of the slider, 0, corresponded to ‘*shared with other species*’ and the top end, 100, to ‘*uniquely human*’. The prosociality scale asked, ‘*Using the slider, please indicate what you think someone who regularly experiences this emotion is like (i.e., how kind are they likely to be?*)’. The bottom end of the slider, 0, corresponded to ‘*extremely unkind*’ and the top end, 100, to ‘*extremely kind*’. The valence scale asked, ‘*Using the slider, please indicate what you think this emotion is like to experience (i.e., how does it make you feel?*)’. The bottom end of the slider corresponded to ‘*extremely negative*’ and the top end to ‘*extremely positive*’.

Emotions were shown in a randomized order for each participant. Alongside rating the emotion terms on a single dimension, participants were asked a binary yes/no question ‘*Have you come across this term before and understand its meaning?’*. This was included to gauge participant’s familiarity with each emotion term. Five attention checks consisted of asking participants, depending on the characteristic being rated, ‘*Please indicate extremely kind/extremely positive/uniquely human*’ or ‘*Please indicate extremely unkind/extremely negative/shared with other species*’ while ‘*attention check*’ was displayed in place of an emotion term.

#### 4.1.3. Procedure

Participants were first shown an example of the scale they would be using to rate each emotion so that they could familiarize themselves with it. The task itself was self-paced with participants rating all of the emotion terms on one of the three dimensions. Under each rating, participants were asked to indicate whether they recognized and understood the emotion term. An optional break was introduced when participants completed 50% of the ratings. The break consisted of a screen with a clear countdown from 60. Instructions declared that this was an opportunity to take a break and that the next rating scale would automatically appear after the 60 s was over. In addition to the emotion ratings, five attention checks were randomly presented. After completing the main task, participants were asked to provide basic demographic information.

### 4.2. Results

A full table of results showing the average ratings of each emotion term on each of the three dimensions, and the percentage of participants who understood its meaning, can be found in the electronic supplementary material.

The 10 emotion terms rated most unique to humans were: disillusion, humble, inspiration, modesty, nostalgia, nostalgic, pessimism, repentance, sense of worth, wickedness. The 10 emotion terms rated least unique to humans were aggression, aggressiveness, distress, exhaustion, fear, fright, pain, protectiveness, scared, suffering. The 10 terms rated most prosocial were caring, compassion, empathy, friendliness, friendship, gratitude, happiness, love, sympathetic, sympathy. The 10 terms rated least prosocial were aggression, aggressiveness, cruelty, hate, hatred, rage, spite, spitefulness, vengefulness, wickedness. The 10 terms rated most positive to experience were bliss, delight, enjoyment, fun, good mood, happiness, happy, joy, love, pleasure. The 10 terms rated least positive to experience were cruelty, depression, hate, hatred, horror, humiliation, rage, suffering, terror, torment.

The availability of rating data for a comprehensive set of emotion stimuli enabled us to explore the relationships between the three dimensions of judgement. To this end, we conducted Pearson’s correlations on the 250 emotion terms using the average ratings for each measure. We found a weak but significant negative correlation between humanness and prosociality ratings, *r* = −0.128, *p* = 0.043, indicating that emotions perceived as more human tend to be viewed as (slightly) less prosocial in nature. There was no significant relationship between humanness and valence ratings, *r* = −0.039, *p* = 0.543. By contrast, prosociality ratings showed a strong positive correlation with valence ratings, *r* = 0.862, *p* < 0.001, suggesting that the perceived kindness of someone expressing a particular emotion is strongly associated with how positive or negative the experience of that emotion is judged to be. However, there were emotions where these two forms of ratings diverged; for example, concern and remorse were rated as prosocial (71%, 61%) but negatively valenced (42%, 38%).

Most emotion terms were familiar to our participants, 223 our of 250 terms were recognized by 90%, or over, of participants. A few terms, however, were less familiar and should probably be avoided in future research with English-speaking participants. These terms were ardour, delectation, disconsolate, exaltation, felicity, forlorn, indignation, lamentation, quandary, rancor/rancour and schadenfreude, all of which had recognition levels below 75%.

This database of emotion stimuli will allow researchers to create large stimulus sets that vary in systematic ways in future research. For example, stimuli that vary in humanness while controlling for prosociality. It also allows us to assess the extent of the confound in previous research.

## 5. Stage 3: investigating the extent to which humanness is confounded with prosociality in infrahumanization research

In stage 3, our main goal was to use the emotion ratings collected in stage 2 to understand the extent to which previous research on infrahumanization inadvertently confounds perceived humanness with prosociality. In particular, we were interested in the percentage of studies in which the secondary emotions used as stimuli were rated as more prosocial than the primary emotions used as stimuli.

For each study investigating infrahumanization of outgroups identified in stage 1, we identified the emotion terms they used to manipulate humanness. We first sought to confirm that they successfully manipulated perceived humanness in the way they intended. In order to do this, we analysed whether the secondary emotions were judged as significantly more human than were the primary emotions. Evidence that a majority of studies successfully manipulated perceived humanness serves as a positive control demonstrating that our ratings procedure is sensitive to variability in emotion stimuli. We then sought to address our main question of interest, the extent to which previous studies inadvertently manipulated perceived prosociality in addition to humanness. To achieve this, we compared the perceived prosociality of secondary and primary emotions used in previous research. A significant difference in prosociality suggests that the stimuli are confounded.

### 5.1. Method

#### 5.1.1. Study selection

Following our pre-registered design, we assessed stimuli from studies that compared emotion attribution with ingroups and outgroups. A list of included and excluded studies, along with reasoning for each decision, is available on OSF/Dryad [26,27]. The total number of included studies was 152, which were drawn from 84 papers.

#### 5.1.2. Planned analyses

There are two particularly common designs in studies of infrahumanization (described below). We varied our analysis strategy accordingly.

*Designs that manipulate humanness*: one common design in the infrahumanization literature is to manipulate the perceived humanity of the emotion terms presented to participants. Preferentially attributing one group (usually an ingroup) secondary emotions to a greater extent than another is thought to demonstrate that they are seen as more human [10,11,19]. In this design, the extent to which the authors successfully manipulated humanness, and inadvertently manipulated prosociality, can be measured with a *t*-test.

For example, Rodríguez-Pérez *et al*. ([19]; studies 1−2) investigated infrahumanization using six secondary emotions (compassion, suspicion, pride, regret, embarrassment and nostalgia) and six primary emotions (surprise, excitement, amazement, tension, restlessness and agitation). In order to assess the effectiveness of the humanness manipulation, we first extracted the humanness ratings for these 12 terms from our emotion rating database. For each of our 200 participants, we then calculated the average humanness rating within the secondary and primary emotion categories, producing a single humanness score per category. Lastly, we conducted a repeated measures *t*‐test to compare the two mean scores (secondary emotions versus primary emotions) across the full sample. If secondary emotions are rated as significantly more human than the primary emotions, then we can conclude that humanness was successfully manipulated.

In order to test whether prosociality was also inadvertently manipulated, we ran another repeated measures *t*‐test (secondary emotions versus primary emotions) with perceived prosociality as the dependent measure rather than perceived humanness. A significant difference in this latter analysis would indicate that the study’s humanity manipulation was confounded with prosociality. We followed this analysis plan for the 66 out of 152 studies which only reported the manipulation of the humanness of their emotion terms.

*Designs that manipulate humanness and valence*: another common design is for researchers to manipulate both the humanity and valence of the emotions. Valence refers to whether the emotions are positive or negative to experience. In this design, the extent to which the authors successfully manipulated humanness, and inadvertently manipulated prosociality, can be measured using a repeated measures ANOVA.

For example, Kteily *et al*. ([37]; studies 2−5) used six secondary emotions, three positive to experience (compassion, tenderness, hope) and three negative to experience (bitterness, regret, shame), as well as six primary emotions, three positive to experience (happiness, pleasure, excitement) and three negative to experience (sadness, pain, rage). In this case, we extracted the humanness ratings for these 12 terms from our emotion database and averaged across our 200 participants’ scores of the secondary positive emotions, secondary negative emotions, primary positive emotions and primary negative emotions. We then conducted a 2 (humanity: secondary primary) × 2 (valence: positive, negative) repeated measures ANOVA with humanity ratings as the measure of interest. A main effect of humanity, ideally combined with no significant interaction, would suggest the researchers successfully manipulated humanity as intended.

In order to assess whether the researchers inadvertently manipulated prosociality, we conducted the same 2 (humanity: secondary, primary) × 2 (valence: positive, negative) repeated measures ANOVA with prosociality as the measure of interest. Evidence for the confound would be revealed by a main effect of humanity on perceived prosociality and/or an interaction between humanity and valence. We followed this analysis plan for the 86 out of 152 studies which reported manipulating both the humanness and valence of their emotion terms.

It is worth emphasizing that, in conducting these analyses, we are not necessarily replicating the analyses authors ran in the original papers. Typically, authors incorporated additional manipulations (such as group membership) leading to more complex factorial designs. In addition, authors often incorporate individual difference variables, for example, symbolic threat. Our principal goal in running these analyses was not to reproduce previous findings, but rather to measure whether the emotion stimuli that form part of wider studies are confounded.

### 5.2. Results

Across 84 records that met our inclusion criteria (see figure 1) we identified and analysed the emotion terms used across 152 individual studies.

#### 5.2.1. Understanding how commonly humanness is successfully manipulated across all studies

We first investigated whether previous studies successfully manipulated the perceived humanness of their emotion terms in the way they intended. We found that the overwhelming majority of studies did. In total, 99.34% of studies (151 out of 152) showed an effect of humanness as intended by the authors. This is indicated by a significant main effect of the emotion manipulation on ratings of humanness. In some cases, there was also a significant interaction between humanness and valence, suggesting that the strength of the humanness manipulation varied between emotions that were positive to experience and emotions that were negative to experience. Only one study [38] showed no significant difference in humanness between primary (*M* = 34.85) and secondary emotions (*M* = 33.90), *t*_199_ = 0.684, *p* = o.494, *d* = o.048, suggesting that humanness was not successfully manipulated in this case. Further details about the analyses, including the simple comparisons for the ANOVAs, can be found in the electronic supplementary material.

Across all studies, the average effect size of the humanness manipulation was assessed in SPSS (5.26) using the separate means, standard deviations and number of participants for ratings of primary emotions and secondary emotions. Using a fixed effects model, results indicated an estimated overall effect size of *d* = 1.58, *Z* = 167.63, *p* < 0.001.

#### 5.2.2. Understanding how frequently humanness is inadvertently confounded with prosociality in the most cited studies on infrahumanization

When seeking to understand the scale of the confound in previous research, we first considered only the most cited papers on infrahumanization included in our set. The 10 most cited empirical papers on infrahumanization according to Google Scholar (as of 23 October 2023) comprise 22 individual studies (see table 1). 95.45% of these studies (21 out of 22) confound humanness with prosociality such that the uniquely human emotions are judged more prosocial than the emotions shared with other species. This is indicated by either a significant main effect of humanness on ratings of prosociality and/or a significant interaction between humanness and valence on ratings of prosociality with at least one simple comparison in the direction we predicted. The remaining study [39] was also found to confound humanness with prosociality, but in a direction opposite of which we predicted (i.e. the primary emotions were judged as being more prosocial than the secondary ones).

**Table 1.** Summary of analyses investigating evidence of successful humanness manipulation and evidence of a prosociality confound for the 10 most cited studies as identified by Google Scholar (as of 23 October 2023).

study	citations	emotion category	emotion terms	successful humanness manipulation?	evidence of prosociality confound?
De Dreu *et al*. [39] study 3	1021	primary positive	affection, pleasure, attraction	Y	Y^a^
		primary negative	fear, exhaustion, pain		
		secondary positive	admiration, hope, surprise		
		secondary negative	embarrassment, contempt, humiliation		
Leyens *et al*. [11] study 1	883	primary	courage, astonishment, fear, exaltation, surprise	Y	Y
		secondary	nostalgia, compassion, pride, remorse		
study 2		primary positive	joy, pleasure, passion	Y	Y
		primary negative	aversion, anger, irritation		
		secondary positive	felicity, delectation, enjoyment		
		secondary negative	melancholia, resignation, disarray		
Kteily *et al*. [37] study 2a	681	primary positive	happiness, pleasure, excitement	Y	Y
		primary negative	sadness, pain, rage		
		secondary positive	compassion, tenderness, hope		
		secondary negative	bitterness, regret, shame		
studies 2b, 3a, 3b, 4 and 5		primary positive	happiness, pleasure, excitement	Y	Y
		primary negative	sadness, pain, rage		
		secondary positive	compassion, optimism, hope		
		secondary negative	bitterness, guilt, contempt		
Demoulin *et al*. [36] study 2	459	primary positive	surprise, attraction, pleasure	Y	Y
		primary negative	anger, disgust, fear		
		secondary positive	compassion, serenity, happiness		
		secondary negative	shame, bitterness, contempt		
Tam *et al.* [40] studies 1 and 2	405	primary positive	surprise, calmness, attraction, enjoyment, caring, excitement, pleasure	Y	Y
		primary negative	pain, fear, anger, fury, panic, fright, suffering		
		secondary positive	optimism, love, passion, elation, nostalgia, admiration, hope		
		secondary negative	humiliation, shame, guilt, disgust, melancholy, disconsolate, disenchantment		
Cuddy *et al*. [12] pilot and study 1	394	primary	confusion, pain, distress, fear, panic, anger, rage	Y	Y
		secondary	grief, sorrow, mourning, anguish, guilt, remorse, resentment		
Vaes *et al*. [14] study 1	380	primary	rage	Y	Y
		secondary	indignation		
study 2		primary	attraction, pleasure, rage, contentment, agitation, anger, excitement, fear	Y	Y
		secondary	admiration, love, compassion, remorse, hope, rancor, regret, passion		
study 3		primary	attraction, fright, desire, pleasure, agitation, excitement, fear, anger	Y	Y
		secondary	admiration, satisfaction, regret, disappointment, love, hope, shame, resentment		
study 4		primary	attraction, desire, excitement, pleasure, agitation, anger, fear, rage	Y	Y
		secondary	admiration, enthusiasm, love, bitterness, regret, disappointment, hope, compassion		
Costello & Hodson [41] study 1	368	primary positive	excitement, joy, pleasure	Y	Y
		primary negative	sadness, fear, rage		
		secondary positive	hope, compassion, friendliness		
		secondary negative	guilt, remorse, shame		
Brown *et al.* [42] study 1	341	primary	enjoyment, pleasure, excitement, affection, happiness, caring, anger, distress	Y	Y
		secondary	hope, good-mood, tenderness, sympathy, optimism, elation, humiliation, sorrow		
Čehajić *et al.* [43] studies 1 and 2	328	primary positive	happiness, euphoria, pleasure, joy	Y	Y
		primary negative	sadness, disgust, anger, fear		
		secondary positive	tenderness, hope, admiration, love		
		secondary negative	remorse, guilt, shame, resentment		

^a^ Denotes the confound being in a direction opposite of what we predicted.

Twenty-four papers in our set have been cited at least 100 times. These 24 papers comprise 53 individual studies: 86.79% (46 out of 53) of these studies contain a confound in the direction we predicted and 5.66% (3 out of 53) contain a confound in the opposite direction.

#### 5.2.3. Understanding how frequently humanness is inadvertently confounded with prosociality across all studies

In the next step of our analysis, we sought to understand how many studies in total inadvertently confound humanness with prosociality.

##### 5.2.3.1. Evidence for the predicted confound

In total, 79.61% of studies (121 out of 152) we assessed showed a confound in the predicted direction as illustrated by either a significant main effect of humanness on ratings of prosociality and/or a significant interaction between humanness and valence on ratings of prosociality with at least one simple comparison in the direction we predicted, secondary emotions rated higher on prosociality compared with primary emotions. Further details about the analyses, including the interactions in the ANOVAs can be found in the electronic supplementary material.

Across all 152 studies, the average effect size of the confound was assessed in SPSS (5.26) using the separate means, standard deviations and number of participants for ratings of primary emotions and secondary emotions. Using a fixed effects model, results indicated an estimated overall effect size of *d* = 0.65, *Z* = 75.14, *p* < 0.001. The confound identified in previous work is thus common within infrahumanization research [22–24]. In the majority of studies, evidence for infrahumanization cannot be clearly separated from evidence for intergroup preference.

##### 5.2.3.2. No evidence of a confound

In 3.95% of studies (6 out of 152) no main effect of humanness was shown on ratings of prosociality and, where ANOVAs were conducted, no interaction between humanness and valence on ratings of prosociality. These six studies were drawn from three papers: [24], [44, study 4] and [45, studies 2b, 3, 4 and 5a]. The stimuli used in these studies appear to show the ideal pattern of results in terms of separating the effects of humanness from prosociality and so might be particularly useful for future research. As a result, we describe the stimuli for these studies in more detail below.

McWaters & Hawkins [24] used pride, remorse and shame as secondary emotions and anger, excitement and pleasure as primary emotions. Unfortunately, however, McWaters & Hawkins [24, p. 113], report that they were unable to analyse the effect of their manipulation on infrahumanization as the reported reliabilities ‘*fell below the recommended level*’. Wohl *et al*. [45] used repulsion and dejection as secondary emotions and rage and sadness as primary emotions. Demoulin *et al*. [44] used admiration, resentment and humiliation as secondary emotions and attraction, anger and sadness as primary emotions.

##### 5.2.3.3. Evidence of a confound in the opposite direction

Surprisingly, some studies showed a confound in the direction opposite to that which we predicted. In 16.45% of the studies we assessed (25 out of 152), primary emotions were rated as significantly more prosocial than were secondary emotions as indicated by a main effect of humanness on ratings of prosociality. These studies are drawn from 15 papers.

From the subset of studies that manipulated valence, 84.88% (73 out of 86) showed a significant interaction between humanness and valence on prosociality. In 29.07% of studies (25 out of 86), positive secondary emotions were rated as less prosocial than were positive primary emotions. In 24.42% of studies (21 out of 86), negative secondary emotions were rated as less prosocial than were negative primary emotions.

## 6. Stage 4: examining the impact of the confound on evidence for outgroup infrahumanization

In stage 3, we devised a method of determining whether or not the stimulus sets used in prior infrahumanization research were confounded by prosociality. Our analysis revealed that the secondary and primary emotion terms that comprised these stimulus sets were often poorly matched on this dimension. Having established the prevalence of this potentially critical oversight in how researchers select their emotion stimuli, we next investigated whether the extent to which a study’s stimulus set is confounded by prosociality influences the size of effects resembling infrahumanization.

To test this question, we collected novel data. We conducted an experiment in which 140 participants who identified as Christian and right-wing were tasked with rating how strongly they felt different emotions are experienced by the group they belong to (Christians) and a group they do not belong to (Muslims). The chosen intergroup context of Christians versus Muslims has been found to produce results consistent with infrahumanization in previous research [15].

The crucial manipulation was the employment of two different sets of emotion stimuli that varied significantly in the degree to which their secondary and primary emotions were matched in perceived prosociality according to our stage 2 participants’ ratings. One of the stimulus sets was taken from study 1 of Vaes *et al*. [46] and was the most confounded with prosociality according to our participants’ ratings. The other stimulus set was taken from Martínez *et al*. [47] and was the least confounded/best matched according to our participants’ ratings.

Infrahumanization theory predicts that participants will attribute secondary emotions more strongly to ingroup members than to outgroup members but show little difference in the attribution of primary emotions. If the confound we have identified has little bearing on the size of this effect, then this pattern will emerge regardless of whether or not the stimulus set is confounded by prosociality. By contrast, our hypothesis is that apparent evidence for infrahumanization is at least partially explained by intergroup preference. Consequently, we expected to observe differential results depending on the stimulus set. We predicted that preferential attribution of secondary emotions to ingroup members would emerge more strongly in the most confounded stimulus set compared with the least confounded stimulus set.

### 6.1. Method

#### 6.1.1. Participants

The sample consisted of 140 adult participants (*M*_age_ = 53.21, s.d._age_ = 15.13), with 69 identifying as female and 71 as male. Participants were pre-screened to include only fluent English speakers located in the UK with a Prolific approval rating ≥80%. They were also required to identify religiously as Christian and politically as right-wing. The reported sample includes two replacement participants for those who failed 50% or more of the attention checks.

The sample size was determined *a priori* from a power analysis conducted in MorePower (version 6.0.4). A minimum *n* of 126 was found to be necessary to detect a 2 × 2 within-subjects interaction of medium effect size ($\eta_{p^{2}}=0.06$) when using an alpha of 0.05 and power of 80%. This was rounded up to 140 to allow for an equal number of participants in each counterbalancing condition. This sample size aligns with previous studies employing similar paradigms and intergroup contexts that were able to detect results consistent with infrahumanization ([22], studies 1a–f; [25]).

The procedure was approved by the University of York Department of Psychology’s Ethics Committee and all methods were performed in accordance with the committee’s guidelines and performed in accordance with the Declaration of Helsinki. Participants were recruited via Prolific (https://www.prolific.ac.uk) and tested via Qualtrics (https://www.qualtrics.com). Participants were reimbursed at an hourly rate of £9.

#### 6.1.2. Selection of emotion stimuli

To identify the stimulus sets that were most confounded and least confounded by prosociality, we used the estimates of effect size from our stage 3 prosociality rating analyses. For each of the 152 studies, we performed a paired *t*‐test contrasting the averaged prosociality ratings of the secondary emotions against the primary emotions. The ‘most confounded’ and ‘least confounded’ stimulus sets were defined as the contrasts which yielded the largest and smallest Cohen’s *d* value, respectively.

The stimulus set identified as being most confounded by prosociality belonged to study 1 of Vaes *et al*. [46]. The secondary emotion terms used in this set (adoration, delight, appreciation, admiration, love, compassion, embarrassment, resentment, nostalgia and concern; *M* = 67.73, s.d. = 6.89) are perceived as more prosocial than the primary emotions terms (attraction, contentment, amusement, astonishment, euphoria, anguish, affliction, anger, sadness and fear; *M* = 48.93, s.d. = 6.28), *t*_199_ = 32.012, *p* < 0.001, *d* = 2.264). The secondary emotions (*M* = 56.25, s.d. = 20.03) were also confirmed to be rated as more uniquely human that the primary emotions (*M* = 38.26, s.d. = 18.85), *t*_199_ = 18.267, *p* < 0.001, *d* = 1.291.

The stimulus set identified as being least confounded by prosociality was study 1 of Martínez *et al*. [47]. The secondary emotions used in this set (love, hope, optimism, contentment, bitterness, melancholy, worry and shame; *M* = 59.39, s.d. = 5.98) are quite well-matched in perceived prosociality with the primary emotions (cheerfulness, fun, tranquillity, enthusiasm, fear, sadness, tension and boredom; *M* = 59.21, s.d. = 6.70), *t*_199_ = 0.377, *p* = 0.706, *d* = 0.027. The secondary emotions (*M* = 57.26, s.d. = 19.40) were also confirmed to be rated as more uniquely human than the primary emotions (*M* = 34.83, s.d. = 20.31), *t*_199_ = 20.606, *p* < 0.001, *d* = 1.457.

#### 6.1.3. Emotion attribution rating task

The emotion attribution rating task was split into four blocks. Each block began with a statement directing participants to consider a target group, either Christians (the ingroup) or Muslims (the outgroup); for example, ‘*In the following questions, please consider the group: people who are Muslims (the group which you do not belong to*)’. The ingroup and outgroup blocks were presented in an alternating order that was counterbalanced across participants.

Within each block, participants encountered a list of questions involving different emotion terms, prompting them to indicate the extent to which they believed the emotions were experienced by the target group (e.g. ‘*How strongly do you think Christians typically feel boredom?’*). Responses were entered using a 100-point sliding scale, with labels of ‘*not at all*’ (0), ‘*somewhat*’ (50) and ‘*very strongly*’ (100).

#### 6.1.4. Design

This experiment deployed a 2 (group: ingroup, outgroup) × 2 (humanness: secondary, primary) × 2 (stimulus set: most confounded, least confounded) within-subjects design.

For half the blocks of the rating task, participants attributed emotions from the most confounded stimulus set, while the other half involved attributing emotions from the least confounded stimulus set (see §6.1.2. for the complete list of included emotions). The secondary and primary emotions within each stimulus set were presented together, with their order randomized. The presentation order of the two stimulus sets was counterbalanced across participants, with half of participants beginning with the most confounded stimulus set, and half beginning with the least confounded stimulus set. One attention check question appeared among the list of emotion attribution questions per block. These took the form of an instruction to enter a particular response for that question (e.g. ‘*please indicate ‘not at all’*, ‘*please indicate ‘very strongly’*).

#### 6.1.5. Procedure

Participants were informed that the study was designed to enhance our understanding of how individuals ascribe emotions to different groups of people. They were given instructions outlining the structure of the study and types of questions they would be asked. After providing consent, participants submitted basic demographic information which was used to verify they met the study inclusion criteria. They were then tasked with completing all four blocks of the emotion attribution task. After completing the main task, participants were asked to rate the ingroup and outgroup on two additional measures included as manipulation checks to confirm the intergroup context was one that we would expect to observe infrahumanization if it occurs. The first was the blatant dehumanization scale [37], in which participants were asked to rate the evolutionary development of each group on a scale marked by visual representations of different stages of human evolution. The second measure was an attitude scale, in which participants were asked to indicate how they felt about each group. The results of both these measures revealed an ingroup preference and are fully reported in the electronic supplementary material.

#### 6.1.6. Analysis plan

In line with our pre-registered analysis plan, we conducted a 2 (group: ingroup, outgroup) × 2 (stimulus set: most confounded, least confounded) repeated measures ANOVA using participants’ humanizing biases as the dependent measure. Humanizing bias scores were quantified by calculating a difference score of the average emotion attribution ratings (mean secondary emotion ratings—mean primary emotion ratings). Positive values are indicative of an increased tendency to attribute secondary emotions over primary emotions. A score of 0 is indicative of the absence of a systematic bias. If the confound with prosociality has little bearing on evidence for infrahumanization, then we would expect to observe a main effect of group membership but no interaction with stimulus set. If apparent evidence for infrahumanization can be at least partially explained by the confound with prosociality, then we will observe a significant interaction between group membership and stimulus set.

We further explored whether there was evidence for preferential attribution occurring for each stimulus set individually by conducting separate 2 (group: ingroup, outgroup) × 2 (humanness: secondary, primary) repeated measures ANOVAs on participants’ mean attribution ratings. If infrahumanization emerges regardless of the stimulus set, then we will observe an interaction between group membership and humanness in both stimulus sets. If apparent evidence for infrahumanization is at least partially explained by intergroup preference, then we will observe a stronger interaction for the most confounded stimulus set compared with the least confounded stimulus set.

We complemented the traditional null-hypothesis significance testing approach with Bayesian analyses. Bayesian paired *t*-tests and ANOVAs were conducted using the default Cauchy priors in JASP (JASP-Team, 2022; *t*-tests: centre = 0, width = 0.707; ANOVAs: fixed effects width = 0.5, random effects width = 1). A Bayes factor (BF_01_) larger than 1, 3 and 10 reflects anecdotal, substantial and strong evidence, respectively, in favour of the null hypothesis. BF_01_ values less than 1, 1/2 and 1/10 reflect anecdotal, substantial and strong evidence, respectively, in favour of the alternative hypothesis [48].

### 6.2. Results

#### 6.2.1. Intergroup emotion attributions

**Figure 2.**
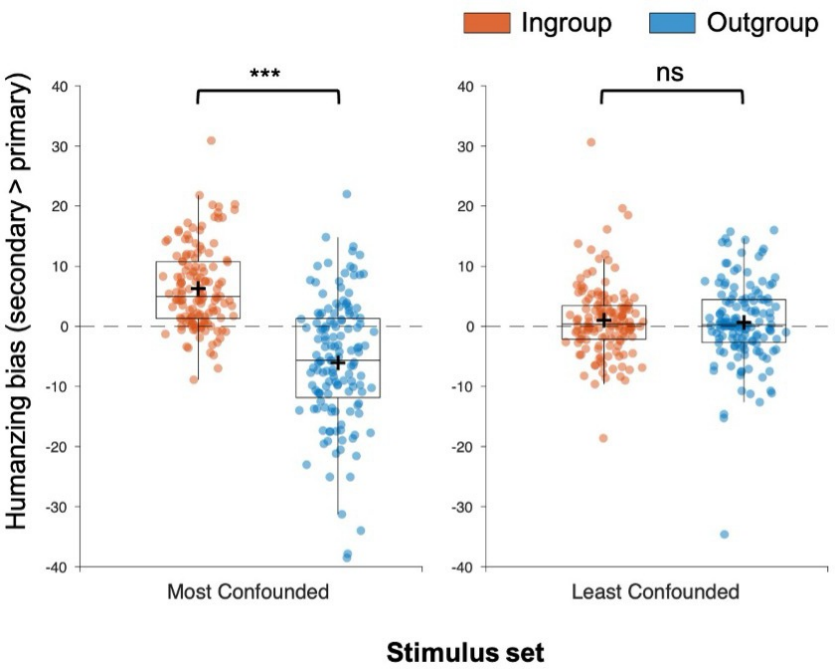
Impact of matched and mismatched stimuli on the presence of preferential attribution of secondary emotions to ingroup members (stage 4). When tasked with attributing emotions known to be poorly matched in perceived prosociality, the results resembled those commonly reported in the infrahumanization literature: participants exhibited a strong bias to attribute uniquely human emotions to ingroup members. However, when attributing emotions known to be approximately matched in perceived prosociality, no such effect was observed. Boxplots denote median and interquartile range, and crosses denote means. *** = *p* < 0.001, ns = *p* > 0.05.

Participants’ humanizing biases were subjected to a 2 (group: ingroup, outgroup) × 2 (stimulus set: most confounded, least confounded) repeated measures ANOVA. The ANOVA revealed a significant main effect of group, *F*_1,139_ = 65.978, *p* < 0.001, $\eta_{p^{2}}=0.322$, BF_01_<0.001, indicating participants exhibited a general tendency to attribute more secondary emotions (relative to attribution of primary emotions) to the ingroup (*M* = 3.64) than the outgroup (*M* = −0.98). We observed no main effect of stimulus set, *F*1,139 = 3.160, *p* = 0.078, *η*_p_^2^ = 0.022, BF_01_ = 2.636, suggesting there was no overall difference in biased attribution of secondary emotions in the most confounded (*M* = 1.84) and least confounded stimulus sets (*M* = 0.83) when ignoring the influence of group. Consistent with our prediction that the expression of a humanizing bias towards the ingroup would emerge most strongly in the most confounded stimulus set, we observed a group × stimulus set interaction, *F*_1,139_ = 64.033, *p* < 0.001, *η*_p_^2^ = 0.315, BF_01_< 0.001. The pattern of results for each stimulus set are shown in figure 2.

To further understand the pattern of results obtained from each stimulus set, separate 2 (group: ingroup, outgroup) × 2 (humanness: secondary, primary) repeated measures ANOVAs were conducted using mean emotion attribution ratings as a dependent measure.

*Most confounded stimulus set*: the ANOVA revealed a main effect of group, *F*_1,139_ = 50.524, *p* < 0.001, *η*_p_^2^ = 0.267, BF_01_< 0.001, whereby participants exhibited a general tendency to attribute more emotions (regardless of type) to the ingroup compared with the outgroup. A main effect of humanness was observed, *F*_1,139_ = 15.629, *p* < 0.001, *η*_p_^2^ = 0.101, BF_01_ = 0.056, suggesting participants were more likely to attribute secondary emotions than primary emotions regardless of target group. We also observed a significant interaction between group and humanness, *F*_1,139_ = 64.033, *p* < 0.001, *η*_p_^2^ = 0.315, BF_01_< 0.001. Pairwise comparisons revealed that the ingroup (*M* = 70.20%, s.d. = 11.01%) were more likely to be attributed secondary emotions than the outgroup (*M* = 60.61%, s.d. = 15.56%), *t*_139_ = 9.978, *p* < 0.001, *d* = 0.843, BF_01_< 0.001. By contrast, no significant difference was found between primary emotion attributions to the ingroup (*M* = 63.92%, s.d. = 12.96%) and outgroup (*M* = 63.21%, s.d. = 13.43%), *t*_139_ = 1.018, *p* = 0.311, *d* = 0.086, BF_01_ = 6.410.

*Least confounded stimulus set*: the ANOVA revealed a main effect of group, *F*_1,139_ = 46.363, *p* < 0.001, *η*_p_^2^ = 0.250, BF_01_< 0.001, whereby participants exhibited a general tendency to attribute more emotions (regardless of type) to the ingroup compared with the outgroup. However, the main effect of humanness did not reach the significance threshold, *F*_1,139_ = 3.776, *p* = 0.054, *η*_p_^2^ = 0.025, BF_01_ = 1.547, suggesting participants did not substantially differ in their attribution of secondary emotions to ingroup/outgroup members compared with primary emotions. Consistent with our prediction that an infrahumanization effect would be smaller in this stimulus set, the interaction between group and humanness was not significant, *F*_1,139_ = 0.300, *p* = 0.585, *η*_p_^2^ = 0.002, BF_01_ = 6.870. Pairwise comparisons revealed that the ingroup (*M* = 66.69%, s.d. = 11.47%) were more likely to be attributed secondary emotions than the outgroup (*M* = 62.07%, s.d. = 13.75%), *t*_139_ = 5.883, *p* < 0.001, *d* = 0.497, BF_01_< 0.001. This was also found to be the case for primary emotions, as there was greater attributions to the ingroup (*M* = 65.68%, s.d. = 12.68%) than outgroup (*M* = 61.43%, s.d. = 13.93%), *t*_139_ = 6.217, *p* < 0.001, *d* = 0.525, BF_01_< 0.001.

## 7. A tool for generating stimuli for future research

Our analyses reveal a prevalent methodological issue in infrahumanization research, wherein stimulus sets inadvertently confound humanness with prosociality (stage 3). Moreover, we have provided demonstration of how this issue probably exerts a substantial negative impact on the reliability of obtained results (stage 4).

To address this problem moving forward in a practical and constructive manner, we have made our emotion rating database available along with an open-source interactive tool. The Emotion Stimuli Selector has been developed to assist researchers in evaluating the extent to which the stimuli they plan to include in their studies are matched or unmatched on the dimensions of humanness, prosociality and valence. Additionally, it provides insights into participant familiarity with the emotion terms.

The primary intention with this tool is to aid with pre-testing stimuli used in infrahumanization research, although we hope it may prove useful to emotion researchers more broadly as well. In most use cases, researchers’ goals will be to generate a stimulus set in which secondary and primary emotions significantly differ in the extent to which they are perceived as uniquely human, while being matched as closely as possible in terms of prosociality and valence. In principle, however, the tool’s use extends beyond this and can accommodate other research goals. For instance, a researcher investigating intergroup attributions of prosocial versus antisocial emotions may seek to confirm differences in perceived prosociality, while maintaining consistency in humanness and valence.

### 7.1. How to use the Emotion Stimuli Selector

Our goal with the Emotion Stimuli Selector was to ensure maximum accessibility and user-friendliness. Consequently, it is available as an open-source Excel workbook downloadable from OSF (https://osf.io/zs2a6/; [26]) and Dryad [27]. We recommend opening the file within Microsoft Excel as this was the primary testing environment (Excel for Microsoft 365 version 2310), although core functions have also been found compatible with other applications, such as Google Sheets.

To generate results, users simply need to select their candidate emotions (up to 10 in each of the two emotion categories) from the drop-down lists at the top of the workbook. The available options encompass the 250 emotions for which we collected rating data (see the electronic supplementary material for a complete list). Note that there are no constraints on emotion selection by category; for example, no restrictions or warnings are issued if a commonly regarded primary emotion is chosen in a secondary emotion slot. Users should exercise caution and possess some *a priori* knowledge to ensure that their chosen emotions accurately represent their designated emotion category.

By default, the two emotion category names are preset to ‘*secondary*’ and ‘*primary*’ emotions. However, there is an option to customize these labels should the user wish to define their categories on the basis of other criteria (e.g. ‘*prosocial*’/‘*antisocial*’ or ‘*positive*’/ ‘*negative*’).

### 7.2. Interpreting the results

The Emotion Stimuli Selector outputs an array of information about the selected emotions on the dimensions of perceived humanness, prosociality and valence. For each dimension, mean ratings (based on samples of *n* = 200) for each individual emotion will be displayed to help identify problematic outliers within the stimulus set. Additionally, summary statistics (means and standard deviations) and bar charts are presented to provide insights into differences in distributions of ratings between the two emotion categories.

The Emotion Stimuli Selector conducts a statistical comparison between the two emotion categories using a process mirroring that used in the stage 3 analyses of this article: a paired-samples *t*‐test (two-tailed) contrasting the averaged ratings within the two emotion categories. It will provide all necessary information to interpret and report the outcome, including *t* statistics, degrees of freedom, *p*-values and effect sizes (Cohen’s *d*). The outcome of the test, in terms of statistical significance (alpha = 0.05) and observed effect size (using guidelines from [49]), will also be provided in text form.

Using a stimulus set used by Costello & Hodson [50] as an example (secondary: hope, empathy, guilt, despair; primary: happiness, excitement, scared, sad), the selector tells us that the emotion categories differ significantly on dimensions of humanness (*p* < 0.001, *d* = 1.81) and valence (*p* < 0.001, *d* = −0.67), but not prosociality (*p* = 0.077, *d* = 0.13). For humanness, the effect size is found to be ‘very large’ whereas there is a ‘medium’ and ‘very small’ effect size for valence and prosociality, respectively. One may interpret this as the set successfully manipulating humanness, being approximately matched on prosociality, but with a potential confound present on valence.

While a statistically significant result may signal successful manipulation/poor matching on a particular dimension, it is important to keep in mind that non-significant findings are not necessarily evidence of stimuli being matched owing to the limitations inherent in null-significance hypothesis testing (i.e. one can only reject the null, not accept it). If matched stimuli is an end-goal, researchers may wish to interpret the non-significant *p*-values in conjunction with effect sizes. Ultimately, rather than us prescribing a universal standard for which all in the field should follow, we leave it to individual researchers to decide their own thresholds of acceptability for stimulus matching based on their specific research goals.

Lastly, the Emotion Stimuli Selector will provide insights into the familiarity of selected emotion terms among our entire participant pool (*n* = 600). Rather than performing a statistical test, the workbook will flag any problematic unfamiliar emotions via colour coding and a warning message, depending on the percentage of participants who indicated a lack of familiarity with the terms. Again, we do not prescribe a fixed rule on what percentage of participants should be familiar with a term for it to be included within a stimulus set.

We hope that this tool will serve as a valuable resource for researchers seeking to assess their stimuli for potential confounds prior to running their studies. Contact information is enclosed within the worksheet and we welcome any feedback, bug reports and feature requests.

## 8. General discussion

Our principal aim in this process was to reassess evidence for infrahumanization theory and to make constructive suggestions for the field going forward. According to infrahumanization theory, individuals attribute secondary emotions (which are perceived as unique to humans) more strongly to ingroup members than to outgroup members [10,11]. Of central importance to infrahumanization theory is the claim that this subtle dehumanization can be distinguished from intergroup preference. As articulated by Leyens *et al*. [11, p. 401], ‘*If the attribution of secondary emotions to the ingroup reflected a mere positivity effect, it would lose its interest and originality…’*. This theory has been extremely influential in the study of dehumanization, and infrahumanization has been reported in a wide range of intergroup contexts [11,15–18].

Recently, however, infrahumanization theory has been criticized for inadvertently confounding evidence for subtle dehumanization with evidence for intergroup preference. Over [51,52] points out that, in previous research, the secondary emotions used as stimuli are sometimes more prosocial (i.e. perceived as more kind) than the primary emotions used as stimuli. In empirical research, Enock *et al*. [22] demonstrated that when prosociality is controlled for, participants attribute more prosocial emotions to their ingroup and more antisocial emotions to their outgroup regardless of whether or not they are perceived as uniquely human.

To date, it is controversial how widespread this confound is. Whereas Enock *et al*. [22] speculate that the confound may be widespread, Vaes [25, p. 2] claims that ‘*the number of studies that has demonstrated outgroup dehumanization over and above intergroup prejudice and outgroup derogation are much higher than Enock et al. [22] would like to admit*’. This stalemate is detrimental to the field. In order for the study of dehumanization to move forward, it is essential to understand which studies inadvertently confound evidence for dehumanization with intergroup preference and which do not.

### 8.1. Summary of findings

We conducted a systematic search of the literature in which we assessed the scale of the confound in 84 papers and 152 individual studies. In the 10 most cited papers of infrahumanization included in our analysis, 95.45% of the studies included in them were confounded in the direction we predicted. In all of the papers cited over 100 times in our set, 86.79% of the studies included in them were confounded in the direction we predicted. Across the 152 studies we addressed in total, 79.61% were confounded in the direction we predicted. Thus, although the confound is not ubiquitous in the field, it is a substantial problem that seriously limits what we can conclude from previous research.

In order to investigate whether previous evidence for infrahumanization can be at least partially explained by intergroup preference, we conducted a novel experiment that employed two stimulus sets used in previous research. Whereas one of these stimulus sets adequately controlled for prosociality, the other badly confounded humanness and prosociality. Evidence consistent with outgroup infrahumanization was present only in the confounded stimuli. These findings therefore provide an initial demonstration of how stimulus sets improperly balanced on this dimension may yield unreliable and diverging results.

In order to help the field move forward, we have created an open-access, interactive database consisting of 250 emotions that we had rated on perceived humanness, prosociality and valence by a substantial number of participants. Creating appropriately matched stimuli is a challenging task (and one that we have not always got right ourselves). We hope that this database will help researchers improve their stimulus sets in at least three ways. First, it will enable researchers to create stimulus sets that vary in terms of perceived humanness while controlling for prosociality and so avoid the confound originally identified by Over [51,52] and empirically investigated by Enock *et al*. [22]. Second, it will allow researchers to base their judgement about which emotions are perceived as uniquely human and which are perceived as shared with other species on more than intuition. Although we found that the overwhelming majority of studies were successful in manipulating the perceived humanness of the emotions, there were some discrepancies between studies. For example, whereas some studies have treated happiness as a primary emotion [37,50,53], others have treated it as a secondary emotion [17,54,55]. Our database will enable researchers to adopt a more systematic approach when selecting emotion terms. Finally, our database offers the opportunity for researchers to increase the number of emotion terms used as stimuli and thus increases the scope for reliability measurement of the different emotion categories.

### 8.2. Significance of the findings

Taken together, this work suggests that previous research on infrahumanization is considerably less convincing than it first appears. Taken in conjunction with other recent findings [22,23], this work suggests that many, perhaps even most, previously reported infrahumanization effects need to be reassessed in light of the methodological problem we have identified.

It is important to emphasize however, that in reporting these results, we are not claiming to falsify infrahumanization theory. It may be that there are some intergroup contexts in which infrahumanization can be reliably observed even when the stimuli are appropriately controlled. Our claim is more modest and more specific—at present we know very little about the prevalence of infrahumanization because a high proportion of previous research confounds attribution of uniquely human emotions with intergroup preference.

It was put to us by a reviewer that because valence and prosociality correlate so highly (*r* = 0.862, *p* < 0.001), controlling for one is more or less equivalent to controlling for the other. In other words, because previous infrahumanization research has often controlled for the valence of the emotion stimuli, it is not necessary to additionally control for prosociality. However, just because two variables correlate highly, it does not follow that they measure the same construct. By analogy, weight and height correlate highly but it would be possible to identify individuals who are short and heavy or tall and light. Conceptually, it is clear that what an emotion is like to experience (valence) is separable from whether or not it is prosocial. Take the example of schadenfreude, or pleasure at another’s expense. By definition this emotion is positive to experience and yet it is antisocial in character. The data we collected in stage 4 demonstrates that controlling for prosociality specifically is crucial. When the perceived humanness of emotion terms is confounded with prosociality, the classic infrahumanization effect emerges. When prosociality is controlled for, evidence for infrahumanization is no longer present.

### 8.3. Limitations

One limitation of our work relates to the types of study we incorporated into our analysis. We considered 152 papers in total, each of which treated the perceived humanness of emotions as a categorical variable. While this is an extremely common approach in the study of infrahumanization, it is not universal. A small number of studies treat the perceived humanness of emotions as a continuous rather than a categorical variable (more specifically: [25,28,56,57]). These studies do not confound emotion humanness with perceived prosociality in the way we describe. It would be valuable for future research to further consider these methods and the ways in which they can be used to test infrahumanization theory. Relatedly, it is interesting to note that, in a minority of studies, there was a confound between prosociality and humanness but in the opposite direction to that which we predicted. It will be important for future research to revisit these stimuli, and replicate the findings from the original papers, in order to understand what these results mean to the field.

Another limitation concerns the infrahumanization experiment that was reported in stage 4. Although we believe these results provide a compelling demonstration of how effects resembling infrahumanization may manifest as a consequence of employing stimulus sets that do not adequately control for prosociality, the generalizability of these findings to the broader field remains uncertain. Despite our efforts to design the experiment to closely resemble a typical infrahumanization study, certain aspects, such as the specific intergroup context of Christians versus Muslims, may render it a less than perfect exemplar. Furthermore, the selection of stimulus sets in this experiment was based on extreme cases incorporating the most confounded and least confounded by prosociality. This raises questions about whether similar differential results would be observed had we used a wider range of stimulus sets.

It is also important to acknowledge the limitations of our emotion database and the ways in which it influences what we can conclude about the prevalence of the confound between prosociality and humanness in previous research. Previous studies on infrahumanization have been run in a variety of languages. It is possible that subtle differences in the meaning of emotion terms between languages, and translational decisions, may influence the extent to which a particular emotion is perceived as uniquely human or prosocial. While it is often assumed that infrahumanization effects generalize to multiple different linguistic and cultural contexts, this remains an important question for future research. While our database will be useful for selecting appropriately controlled stimuli for English language participants, it would be useful to develop databases in other languages and cultural contexts in order to study infrahumanization effects in a range of contexts. It would also be useful to broaden the Emotion Stimuli Selector so that it includes a wider range of judgements about the emotions incorporated into it. In this work, we limited our focus to humanness, valence and prosociality. In principle, however there may be other dimensions along which emotions vary that have theoretically significant implications for our understanding of infrahumanization theory. For example, the frequency with which different emotion terms are used in natural language and intensity [36]. It will be useful for future research to identify dimensions of interest and formulate ways to measure their impact.

### 8.4. Future directions

The chief priority for future research must be to return to classic findings in this field and determine which findings remain robust when prosociality is controlled for. Only once such a re-evaluation has taken place, can we hope to understand the role of infrahumanization in understanding the psychological mechanisms underlying discrimination.

More broadly, it will be important to more deeply consider how people understand the concept ‘human’. Some researchers have argued that the concept ‘human’ is inextricably linked to positive evaluation [7]. However, this view has proved controversial [51,58]. In our dataset, the extent to which emotions were judged to be uniquely humans was negatively correlated with the extent to which they were deemed prosocial (*r* = −0.128). While these results should be interpreted with caution owing to limitations of our database, they suggest that these dimensions of judgement might be less closely related than previously assumed. Carefully delineating lay concepts of ‘humanness’ across cultures remains a crucial priority for future research.

### 8.5. Conclusions

Taken together, our results suggest that the overwhelming majority of previous research on infrahumanization confounds evidence for subtle dehumanization with intergroup preference. Close to 80% of the findings we assessed proved problematic, seriously limiting what we can conclude about the cognitive mechanisms underlying discrimination. Without a better grasp of the ways in which human psychology contributes to negative outcomes for marginalized groups, we cannot hope to contribute to research-led efforts to reduce this pressing social problem. We have offered an open-source database and analysis tool which we hope will provide the basis for more reliable research in this field in the future.

## Data Availability

Data supporting each stage of the project including the Emotion Stimuli Selector can be found on the Open Science Framework (https://osf.io/zs2a6/; [26]) and Dryad [27]. Supplementary material is available online [59].

## Footnotes

^1^
The reasons for these exclusions were as follows: three studies did not investigate infrahumanization of human groups ([32], studies 1 and 3; [33], study 1), one study was not the required article type [34], one study controlled for emotion prosociality ([22], study 3) and one study did not meet the required experimental design criteria [35].
